# Effects of Temperature on the Evolution of Yield Surface and Stress Asymmetry in A356–T7 Cast Aluminium Alloy

**DOI:** 10.3390/ma14247898

**Published:** 2021-12-20

**Authors:** Elanghovan Natesan, Johan Ahlström, Stefan Eriksson, Christer Persson

**Affiliations:** 1 Department of Industrial and Materials Science, Chalmers University of Technology, 41296 Gothenburg, Sweden; johan.ahlstrom@chalmers.se (J.A.); christer.persson@chalmers.se (C.P.); 2 Volvo Car Corporation, Analysis and Verification, 40531 Gothenburg, Sweden; stefan.a.eriksson@volvocars.com

**Keywords:** aluminium, steel, mechanical behaviour, fatigue, stress asymmetry, isotropic hardening

## Abstract

As the electrification of vehicle powertrains takes prominence to meet stringent emission norms, parts of internal combustion engines like cylinder heads are subjected to an increased number of thermal load cycles. The cost-effective design of such structures subjected to cyclic thermo-mechanical loads relies on the development of accurate material models capable of describing the continuum deformation behaviour of the material. This study investigates the effect of temperature on the evolution of flow stress under cyclic loading in A356-T7 + 0.5% Cu cast aluminium alloy commonly used in modern internal combustion engine cylinder heads. The material exhibits peak stress and flow stress asymmetry with the stress response and flow stress of the material under compressive loading higher than under tension. This peak and flow stress asymmetry decrease with an increase in temperature. To compare this stress asymmetry against conventional steel, cyclic strain-controlled fatigue tests are run on fully pearlitic R260 railway steel material. To study the effect of mean strain on the cyclic mean stress evolution and fatigue behaviour of the alloy, tests with tensile and compressive mean strains of +0.2% and −0.2% are compared against fully reversed (R_ε_ = −1) strain-controlled tests. The material exhibits greater stress asymmetry between the peak tensile and peak compressive stresses for the strain-controlled tests with a compressive mean strain than the tests with an identical magnitude tensile mean strain. The material exhibits mean stress relaxation at all temperatures. Reduced durability of the material is observed for the tests with tensile mean strains at lower test temperatures of up to 150 °C. The tensile mean strains at elevated temperatures do not exhibit such a detrimental effect on the endurance limit of the material.

## 1. Introduction

Cast aluminium and cast iron have been the principal choice of material for casting internal combustion engine (ICE) cylinder heads. In recent years, aluminium has become the preferred material owing to its high thermal conductivity, higher specific strength and stiffness and ability to be cast to complex three-dimensional geometries and close tolerances [1,2]. Pure aluminium is soft, and hence, various alloying elements are added to modify and enhance the final properties of the alloy to suit the various applications. The aluminium–silicon alloy system is widely preferred for casting cylinder heads owing to their reduced production costs, better castability, suitable corrosion and mechanical properties, to list a few benefits. Magnesium, zinc, copper, manganese and iron are often added in varying amounts to the aluminium–silicon alloy, and all of them play different roles in tailoring the properties of the alloy system. The mould-sticking behaviour of the molten alloy is often improved with iron but at the cost of reduced material ductility. This is often counteracted with additions of manganese. The alloys designated A319, A356 and A357 are some of the more commonly used for casting cylinder heads of light-duty engines [3]. The A356 family of alloys have taken prominence in recent years owing to the precipitate strengthening effects of Mg_2_Si in the alloy, offering better creep strength at higher operating temperatures [4]. With an increase in cylinder pressures and specific power, alloys with higher copper content like A356 + 0.5% Cu are increasingly used in cylinder heads [5,6,7,8].

Hot components of an internal combustion engine (ICE) like cylinder heads, exhaust manifolds, etc., are subjected to cyclic thermal loads during their lifecycle. The components can experience severe temperature transients, say, from −30 °C during a cold start in winter months to operating temperatures of more than 230 °C in hot parts of the power train systems [8,9,10]. Such start–operate–stop thermal cycles induce low cycle thermo-mechanical fatigue failures in sections that are particularly susceptible to cracking, such as the valve bridge areas in cylinder heads [11,12,13]. Current trends in the automotive landscape rely on hybrid powertrains using downsized engines with high specific power. The internal combustion engines, part of hybrid powertrains, have increased demands on material durability owing to such start–operate–stop thermal cycles, coupled with an associated rapid approach to peak power compared to traditional combustion engine powertrains. This increased number of thermal load cycles increases the susceptibility of premature thermo-mechanical cracks in vulnerable sections of the cylinder head. Designing cylinder heads with predictable durability is a vital step of the powertrain development process to meet the service life targets [11,14]. To numerically predict the thermo-mechanical fatigue life of such thermally loaded components, engineers rely on numerical models that can model the cyclic load–response of the material and a suitable fatigue criterion to estimate the life of the structure [9,15,16]. Metallic materials, when subjected to cyclic loads, often exhibit a change in the yield strength, the so-called isotropic hardening, which is a function of temperature, strain rate, etc., for a given material [17,18]. For structural materials subjected to cyclic thermal loads like cylinder heads, a good understanding of the evolution of yield surface at various temperatures is imperative for the development of suitable material models in the computer-aided design (CAD) development process.

Mercer et al. [19], while studying the cyclic deformation behaviour of different aluminium alloys, observed a strong peak stress asymmetry in dispersion strengthened IN-905XL, IN9052 alloys and in the precipitation strengthened AA7075-T6 alloy. The peak stresses observed under compression was higher than in tension for a fully reversed strain-controlled cyclic loading. Ma et al. [20] observed a similar compression-tension peak stress asymmetry in poly crystalline copper and ascribed this difference to differential constraints in tension and compression induced by inhomogeneous deformations between the different phases in the material. Several studies have been reported on the peak stress asymmetry observed in various aluminium alloys under cyclic loading [21,22,23].

Ahlström et al. [18,24] studied the effect of temperature on the cyclic yield strength evolution at various temperatures in R7T railway wheel steel and observed that temperature has a dramatic influence on both the magnitude and cyclic evolution of the yield strength. They observed about 70% reduction in the cyclic yield strength levels at 600 °C when compared to the room temperature values.

Azadi [25] studied the effect of mean strain on the fatigue behaviour of A356 and A357 aluminium alloys and observed no significant effect of the mean strains on the number of cycles to failure using strain-controlled tests with high temperature isothermal or out of phase thermo-mechanical load cycles in A356 alloys. The strain ratios and mean strains, however, were reported to affect the number of cycles to failure of the A357 alloy, with a reduction in the fatigue life observed with an increase in tensile mean strains. In studies under stress-controlled loading by Houria et al. [26] examining the effect of mean stress on the multiaxial fatigue behaviour of A356-T6 alloys, the authors observed that tensile mean stress had a detrimental effect on the number of cycles to failure. This observation was more pronounced under tension than under torsion or combined tension-torsion loading conditions. The mode of load-control, namely, stress or strain control, seems to have a pronounced impact on the determination of the number of cycles to failure of the material.

Very few studies have been reported on the effect of temperature on stress and cyclic yield strength asymmetry in aluminium alloys. Similarly, there is a dearth of relevant literature studying the effects of mean strains on the stress response and fatigue behaviour of the new generation of A356 alloys with added copper. To further highlight the differences in the cyclic stress development and yield strength asymmetry, cyclic strain-controlled tests with equivalent loads are run on R260 pearlitic steel commonly used in the railway industry.

## 2. Materials and Methods

### 2.1. Sample Extraction and Sample Preparation

**Figure 1:**
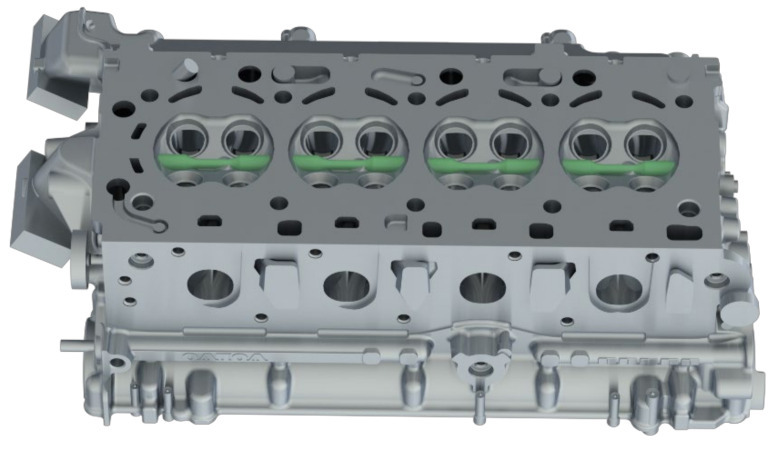
Illustration of test specimen extraction from the highly loaded valve bridge areas of a cylinder head.

**Figure 2:**
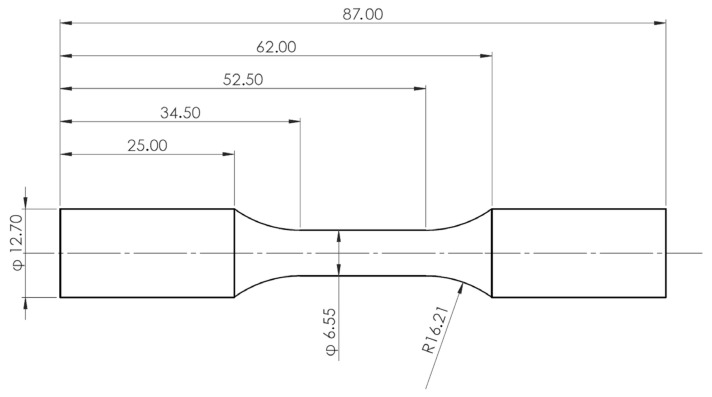
Geometry of the test specimens manufactured in accordance with the ASTM E606/E606M test standard. All the presented dimensions are in mm.

The samples for testing have been extracted from gravity die cast cylinder heads of Volvo Cars’ inline 4 cylinder, VEP-4 series of petrol engines. The samples are extracted so that the test volume of the material lies within the valve bridge volumetric area of the cylinder heads, as illustrated in Figure 1. This valve bridge section is susceptible to thermo-mechanical fatigue crack initiations [16,27,28] and is the area of interest in this study. The extracted material is then machined in accordance with the recommendations from the ASTM E606/E606M standard for strain-controlled fatigue tests [29], and the geometry of the specimens used for testing in this study is presented in Figure 2. The machined samples were subsequently ground and polished sequentially to obtain a mirror surface finish. This helps mitigate the influence of surface irregularities in the fatigue life determination of metallic materials.

### 2.2. Material

#### 2.2.1. Chemical Composition and Microstructure

The chemical composition of the tested alloy can be ascribed to the broader group of the A356 family of cast aluminium alloys. The average measured the chemical composition of the tested A356-T7 + 0.5% Cu alloy, indicating the major alloying elements, is summarised in Table 1. The chemical composition measurements were made using a modified ASTM E1251 test method employing optical emission spectrometry [30,31].

**Figure 3:**
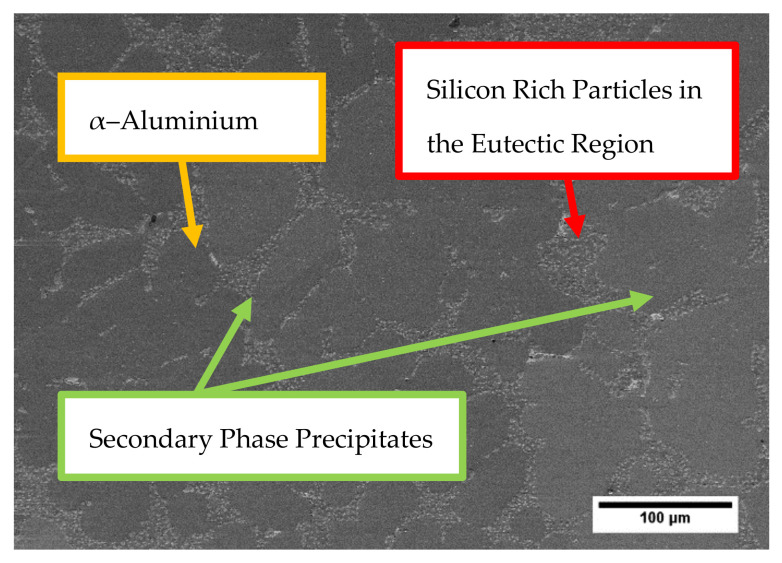
A scanning electron microscopic (SEM) image showing the different phases present in the tested A356-T7 + 0.5% Cu aluminium alloy.

A scanning electron microscopic image of the A356-T7 + 0.5% Cu alloy taken using a Zeiss LEO 1550 Scanning Electric Microscope equipped with a Centaurus back scattered electron detector is presented in Figure 3. The image shows the presence of the major α-aluminium matrix with the silicon particles formed from eutectic solidification and the secondary phase precipitates. The phases are all distinguished by varying shades of grey and are labelled with some representative regions indicated by the arrows.

#### 2.2.2. Manufacturing Process and Heat Treatment

The cylinder heads were produced using the gravity tilt casting method. The alloy melt was maintained in the temperature range 690–710 °C. Titanium was added to refine the grain size, while strontium was used in the melt for eutectic modification. To degas the melt, a spinning 220 mm graphite rotor at 300 rpm was used by simultaneously pumping in nitrogen into the melt. The die temperature was maintained between 200 and 240 °C. The combustion chamber side of the cylinder head was water cooled to obtain a finer dendritic spacing through faster directional solidification. The final heat treatment process started with solutionising the cast at 530 °C for about 3 h. The cast was then quenched in the air. The final artificial “T7” ageing process involved heating the cast to temperatures of 200–230 °C for up to 5 h to achieve high strength, ductility and microstructural stability.

#### 2.2.3. Railway Steel Used for Investigation

To highlight the differences in the yield strength evolution and the stress asymmetry in aluminium alloys with that of steel, a R260 grade fully pearlitic rail steel was tested. The measured chemical composition is summarised in Table 2, and the material and microstructure of the alloy are further detailed in [32].

### 2.3. Test Plan

1. To study the effect of mean strain and temperature on the mean stress relaxation and number of cycles to failure of the A356-T7 + 0.5% Cu alloy, strain-controlled cyclic loads were imposed with mean strains of +0.2%, 0% and −0.2% at room temperature, 150, 200 and 250 °C. All the above tests were run with a total strain amplitude of 0.4% by applying the strain-controlled load in a triangular wave form and at a strain rate of 1% s^−1^, obtained by setting the corresponding test frequency.
2. To develop the cyclic stress–strain curve of the tested aluminium alloy, an additional completely reversed strain-controlled test was run with a total strain amplitude of 0.3% at an identical strain rate of 1% s^−1^.
3. To compare the yield stress and cyclic stress–strain asymmetry of the aluminium alloy with that of general steel, R260 pearlitic steel specimens extracted from rail heads were tested using completely reversed strain-controlled tests at room temperature with total strain amplitudes of 0.3 and 0.4% and at identical test strain rates of 1% s^−1^. A similar strain-controlled, triangular wave load application was used for testing the steel specimens as well to maintain consistency and to enable comparison.

### 2.4. Test Equipment and Set-Up

#### 2.4.1. Equipment Used

All the tests were conducted using an Instron 8501 uniaxial servo-hydraulic machine. The servo-hydraulic machine was equipped with a 1 kHz data acquisition system to sample the strain, axial force and displacement measurements. The high-temperature isothermal tests were executed by using a convection-based Instron 3119-407 test chamber encapsulating the test set-up. Two K-type thermocouples located inside the high-temperature chamber were used for feedback and temperature control. Two types of extensometers were used for the experimental strain measurements depending on the temperature and are detailed in Table 3. The temperatures of the test specimens were recorded using a K-type thermocouple mounted on the specimen surface in the test volume in between the extensometer blades, and the maximum variation of the measured temperature was observed to be within the extensometer limits of error specified at ±2.5 °C.

#### 2.4.2. Thermal Stabilization

To eliminate the interference from thermal gradients in the test set-up on the test measurements, the test set-up was heated to the target temperature and allowed to stabilize for three hours. The tests were started once the thermal gradients and expansion of the test set-up had stabilized.

### 2.5. Analysis of Experimental Results

To study the isotropic hardening behaviour of the alloy, it is necessary to study the evolution of the cyclic yield strength with accumulated plastic strain [33]. The definition and determination of the terms ‘cyclic yield strength’ and ‘accumulated plastic strain’ are described in the sections below.

#### 2.5.1. Nomenclature

**Figure 4:**
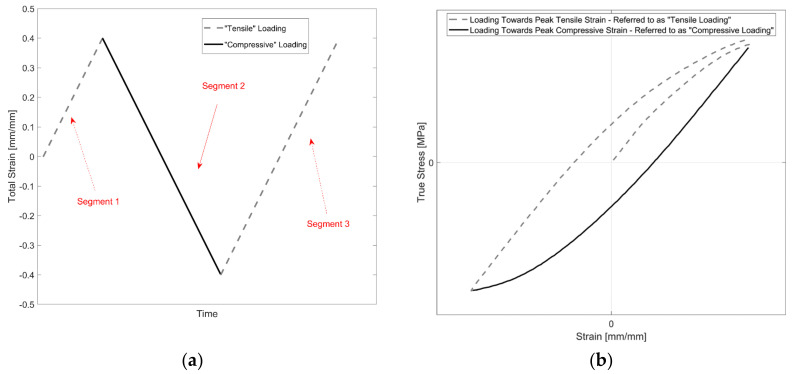
Illustration of nomenclature used for the loading segments (segment contains data for each strain load reversal): (**a**) segment nomenclature for the applied strain-controlled load; (**b**) illustration of the corresponding stress response for each strain load segment.

For brevity, each strain load reversal is named a ‘Segment’ and is illustrated in Figure 4a. For example, for a completely reversed strain-controlled test with a total strain amplitude of 0.4%, the initial loading to the maximum tensile strain from 0% to +0.4% is named Segment 1, the subsequent load reversal from +0.4% to −0.4% is named Segment 2, and so on.

To distinguish the evolution of yield strength with sequential load reversals going towards the tensile and compressive maximum strains during a cycle, the loading towards the tensile maximum strain is named ‘Tensile Loading’, and the loading towards the compressive peak strain from the tensile peak strain is referred to as ‘Compressive Loading’ and is illustrated for the first three segments in Figure 4b.

#### 2.5.2. Cyclic Yield Strength Determination

**Figure 5:**
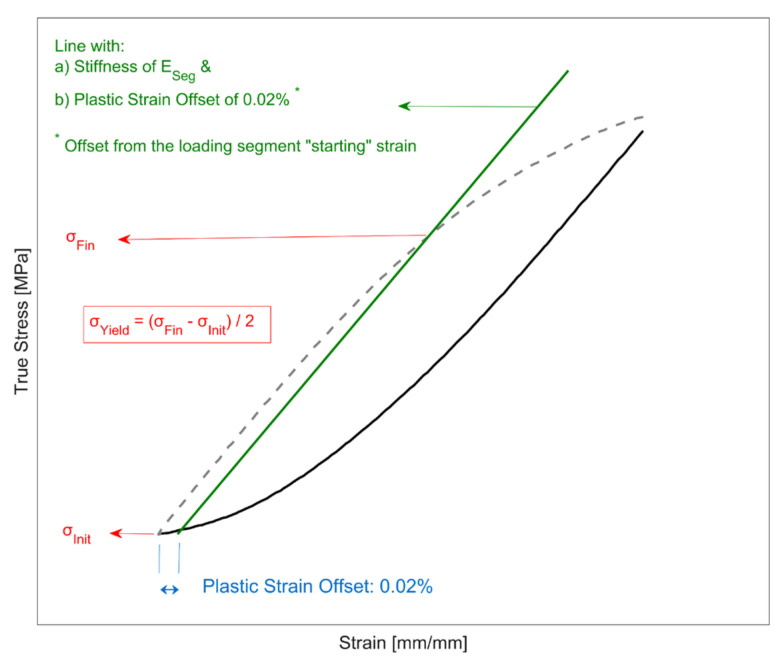
Illustration of how the cyclic offset yield strength is determined for the individual loading segments (image not drawn to scale).

To estimate the yield strength of each loading segment, a small plastic strain offset of 0.02% towards the centre of the hysteresis loop was used to identify the offset yield strength and the methodology is illustrated in Figure 5. The choice of the tangent modulus has a significant influence on the quantitative determination of the cyclic yield strength evolution. The offset line had a slope equal to the linear elastic stiffness estimated for each of the loading segments being evaluated. The linear stiffness of each loading segment was approximated using a two-step iterative process.

Step 1—Use a smaller sample size of the initial elastic data to get a preliminary approximation of the linear elastic stiffness and the offset yield strength of the loading segment being evaluated.

1. Estimate a preliminary stiffness (*E_Prel_*) using either of the following steps depending on the loading segment:
  i. For the first 2 segments, data points from when the elastic loading/unloading commences up to a stress response of 60 MPa are used for the preliminary stiffness approximation. The choice of 60 MPa was dictated by the necessity to have a sufficient collection of sampling points while limiting the extraction of data points from just the elastic loading/unloading of the segment stress–strain curve.
  ii. For all the subsequent loading segments, by using the stress–strain data of the current loading segment for the preliminary stiffness approximation with the data sampled for the evaluation limited to half the yield strength of the corresponding tensile/compressive loading of the prior cycle.
2. Estimate a preliminary yield strength using a plastic offset strain of 0.02% and the estimated preliminary stiffness (*E*_Prel_). The stress range is identified from the initial stress from where the elastic loading/unloading begins and the stress value at the point of intersection of the offset line and the segment’s stress–strain curve. The preliminary yield strength (*σ*_Prel_) is estimated as half the stress range estimated.

Step 2—A revised sample size of the elastic region of the segment stress–strain data is now sampled using the preliminary yield strength (*σ_Prel_*) estimated in the previous step for a final re-evaluation of the segment stiffness and the yield strength.

1. A revised sample size of the segment’s stress–strain data is used for the final linear elastic stiffness (*E_Seg_*) evaluation, with the data limited to half the preliminary yield strength evaluated in the previous step.
2. The final yield strength is determined using a plastic offset strain of 0.02% from the elastic unloading/loading starting point using the re-evaluated segment linear elastic stiffness (*E_Seg_*). Half the difference between the initial stress data point (*σ_Init_)* and the point of intersection of the new offset line (*σ_Fin_*) gives the final cyclic yield strength estimation for the segment and is illustrated in Figure 5.

#### 2.5.3. Calculation of Accumulated Plastic Strain

In cyclic loading, the evolution of the yield strength is often associated with the accumulation of plastic strains in the material [34]. Once the yield strength of the material is exceeded for each loading segment, the material accumulates plastic strains. In cyclic loading, the accumulated plastic strain is a positive scalar that increases incrementally with every plastic loading regardless of the loading direction in the material [35]. For every load reversal, the deformation proceeds from elastic unloading to elastic-plastic loading. For each loading segment ‘n’, using a linear approximation for the elastic modulus, the elastic component of the deformation is removed from the total deformation to obtain the residual plastic strains. Such plastic strains are accumulated from the initial loading till the specimen rupture under cyclic loading together with the concurrent evolution in the yield strength. The data are cut off when a major crack has been established and when there is a corresponding reduction in the tensile stresses. To avoid including the accumulation of plastic strains after a major crack has been established at the end of the life, the cut-off point is identified by observing when the peak tensile stress drops below 75% of the peak stress developed at a stabilized-reference cycle, taken to be the 25th cycle in this case. The mathematical formulations are summarised below:

(1) $$\varepsilon_{Seg_{n}\_Plast}=\varepsilon_{Seg_{n}\_Total}\;-\;\frac{\sigma_{Seg_{n}}}{E_{Seg_{n}}}$$

where $\varepsilon_{Seg\_Plast}$ is the segment plastic strain, $\varepsilon_{Seg\_Total}$ is the segment total strain, $\sigma_{Seg}$ is the segment stress and $E_{Seg}$ is the segment linear elastic stiffness approximation determined as described above. Once the plastic strains in the segment have been evaluated using the expression above, the maximum plastic strain induced in the current loading segment, $\varepsilon_{Max\left(Seg_{n}\_Plast\right)}$, is then extracted using the formulation below:

(2) $$\varepsilon_{Max\left(Seg_{n}\_Plast\right)}=Max(\varepsilon_{Seg_{n}\_Plast})$$

**Figure 6:**
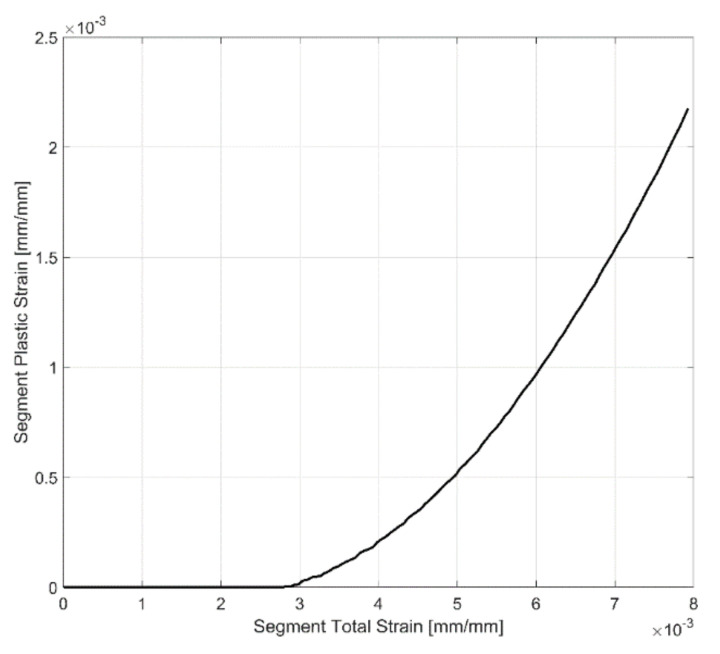
Evolution of segment plastic strain.

**Figure 7:**
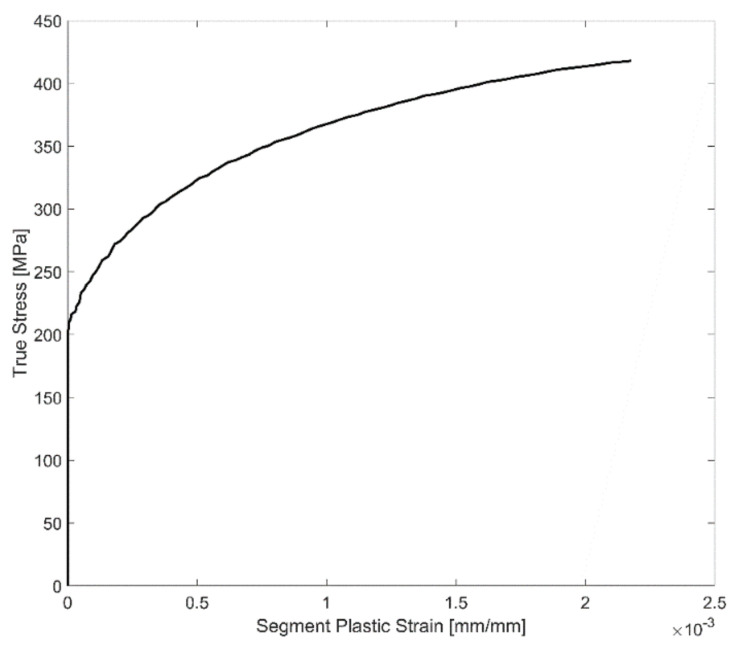
Segment plastic strain vs. stress response.

The accumulated plastic strain after segment ‘n’ (${\bar{\varepsilon }}_{Plast\_After\;Seg_{n}\;}$) is the sum of the accumulated plastic strain after segment ‘n−1′ (${\bar{\varepsilon }}_{Plast\_After\;Seg_{n-1}\;}$) and the maximum plastic strain in the loading segment ‘n’ as shown below:

(3) $${\bar{\varepsilon }}_{Plast\_After\;Seg_{n}\;}\;={\bar{\varepsilon }}_{Plast\_After\;Seg_{n-1}\;}+\varepsilon_{Max\left(Seg_{n}\_Plast\right)}$$

and with the accumulated plastic strain when the cyclic loading begins, i.e., before segment 1 is obviously zero. The evolution of plastic strain with the increase in deformation for a sample segment is presented in Figure 6, and the corresponding stress response with increasing plastic strains in the segment is presented in Figure 7.

## 3. Results

### 3.1. Yield Strength Evolution

**Figure 8:**
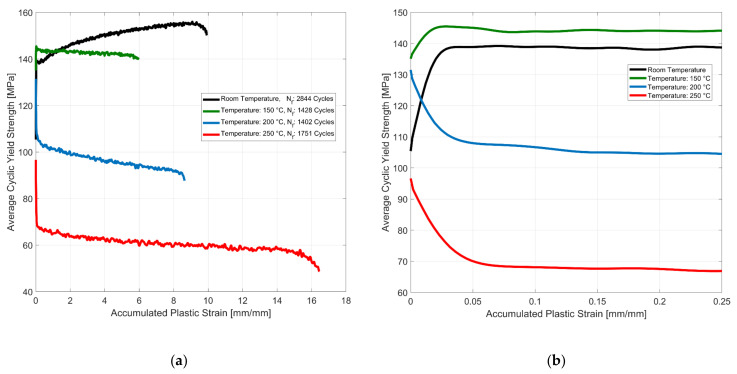
(**a**) Effect of temperature on isotropic hardening, *ε_Amp_*: 0.4%, *R_ε_*: −1; (**b**) magnified plot indicating the initially exponential saturation behaviour of the cyclic yield strength evolution.

The evolution of the average tensile and compressive cyclic yield strength with the accumulation of plastic strain is presented in Figure 8. The material exhibits a non-linear change in the size of the yield surface with the accumulation of plastic strains. For the test at room temperature, the material exhibits an initial exponential increase in the size of the yield surface. The isotropic hardening of the material takes place with further cycling and accumulation of plastic strains but at a lower hardening rate. At 150 °C, the material exhibits an initial steep isotropic hardening with the accumulation of plastic strains. However, with subsequent cyclic loading, the material shows a marginal reduction in the size of the yield surface at a constant rate with the accumulation of plastic strains. The higher yield strength at 150 °C when compared to the estimated yield could be attributed to the scatter in the mechanical properties often observed in A356 cast aluminium alloys and that which has been reported more extensively in our previous work [36,37]. At the higher tested temperatures of 200 and 250 °C, the material exhibits an exponential isotropic softening behaviour with a steeper initial reduction of the size of the yield surface followed by a constant slower reduction with the continued plastic cycling of the material.

### 3.2. Yield Strength Asymmetry

**Figure 9:**
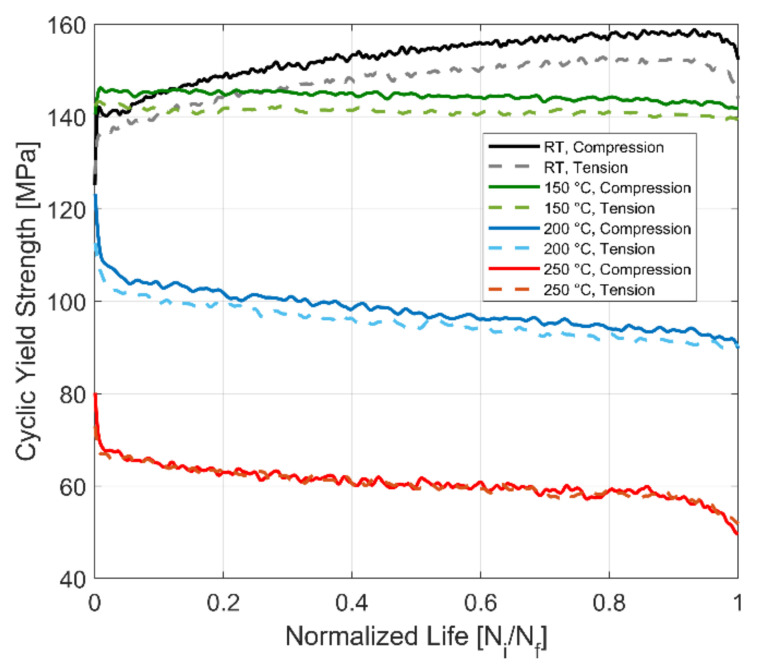
Effect of temperature on yield strength asymmetry, *ε_Amp_*: 0.4%, *R_ε_*: −1.

The evolution of the tensile and compressive yield strength of the A356-T7 + 0.5% Cu at room temperature, 150, 200 and 250 °C is presented in Figure 9 for completely reversed strain-controlled fatigue tests with a total strain amplitude of 0.4%. The yield strength for the compressive loading is higher than that of the tensile loading for all the tested temperatures. The difference in the yield strength between tension and compression is about 10 MPa for the room temperature test and decreases with increase in temperature. At the highest tested temperature of 250 °C, there is no discernible difference in the yield strength between tensile and compressive loading.

**Figure 10:**
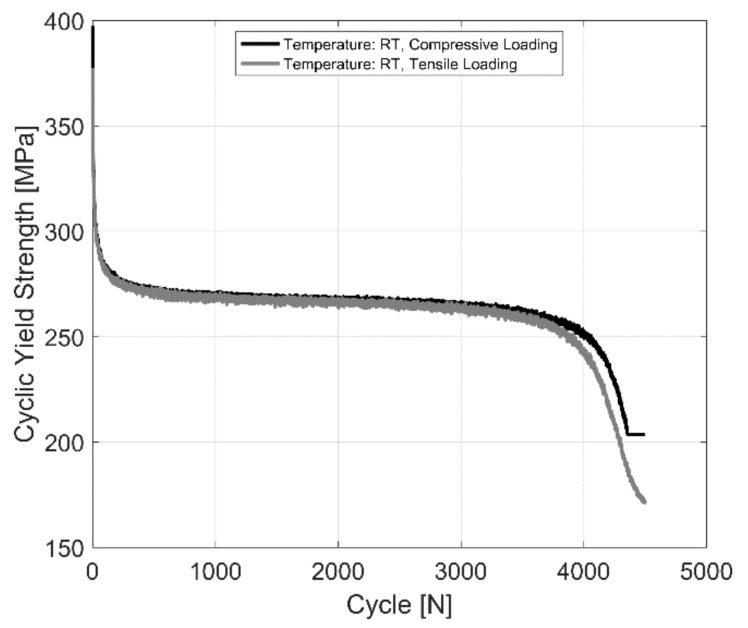
Yield strength evolution in R260 pearlitic steel, *ε_Amp_*: 0.4%, *R_ε_*: −1.

**Figure 11:**
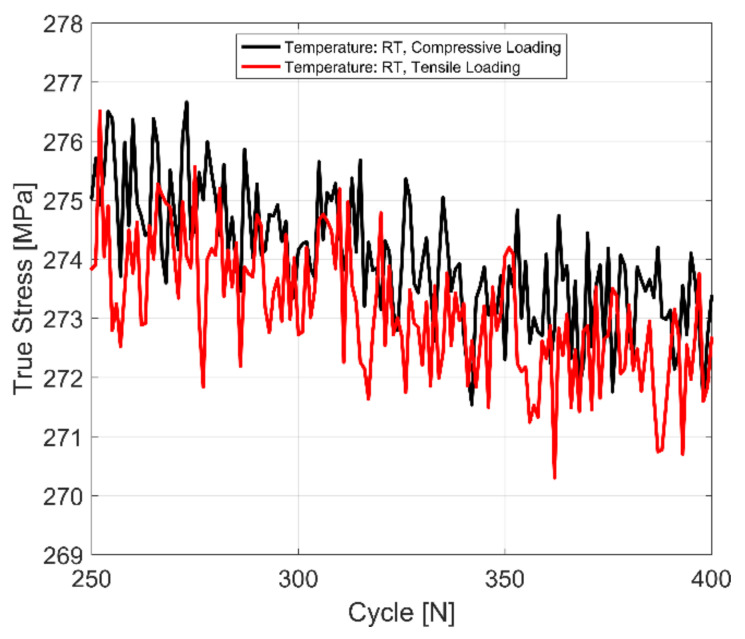
A magnified image of the yield strength in R260 pearlitic steel under tensile and compressive loading, *ε_Amp_*: 0.4%, *R_ε_*: −1.

The tensile and compressive yield stress evolution behaviour of a fully pearlitic R260 railway steel at room temperature for completely reversed, strain-controlled cyclic tests with a total strain amplitude of 0.4% is presented in Figure 10 for comparison. A magnified image of the yield stress under tension and compression is further presented in Figure 11. As can be observed, the R260 railway steel exhibits no significant yield strength asymmetry upon load reversal.

### 3.3. Peak Stress Asymmetry and Cyclic Behaviour

#### 3.3.1. Peak Stress Levels

**Figure 12:**
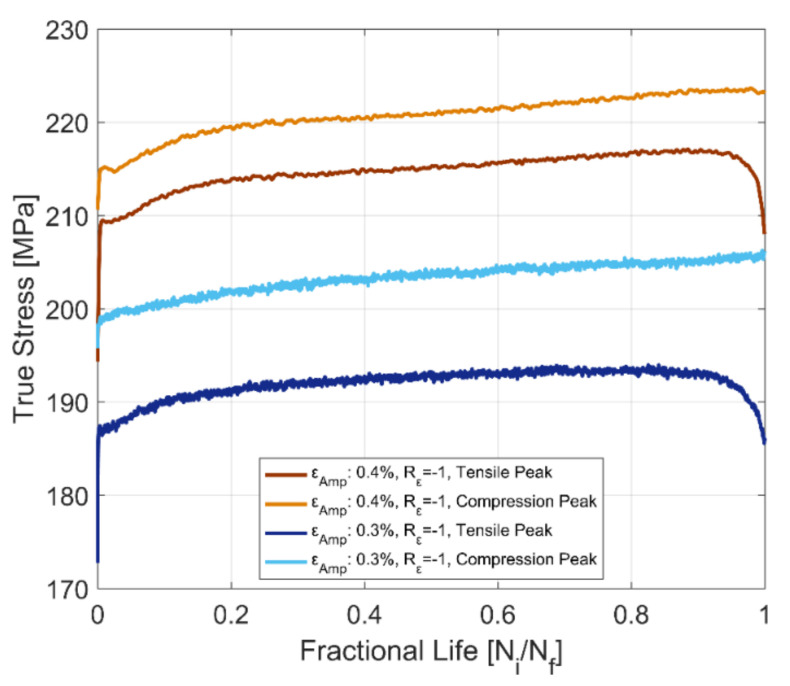
Evolution of tensile and compressive stress peaks, A356-T7 + 0.5% Cu cast aluminium alloy.

**Figure 13:**
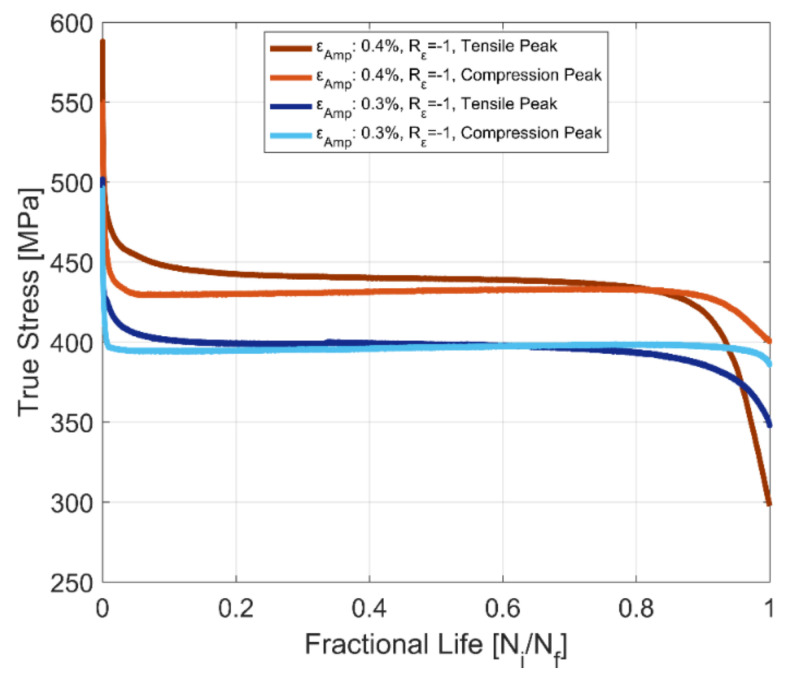
Evolution of tensile and compressive stress peaks, R260 pearlitic steel.

The tensile and compressive peak stress development at room temperature for fully reversed, strain-controlled fatigue tests, with two different total strain amplitudes of 0.3 and 0.4% of the tested A356-T7 + 0.5% Cu cast aluminium alloy and the fully pearlitic R260 railway steel is presented in Figure 12 and Figure 13. The peak compressive stress developed for the aluminium alloy is higher than the peak tensile stress developed under strain-controlled cyclic loading. For the pearlitic steel, however, we observe an opposite response, where the peak tensile stresses developed are higher than the peak compressive stresses for both strain amplitudes at room temperature. The peak tensile stress, however, drops rapidly after about 80% of the life of the specimen and continues decreasing until fracture. This can be attributed to the development of a major crack in the test volume, which has a deleterious effect on the peak tensile stresses but not on the peak compressive stress response.

#### 3.3.2. Cyclic Stress–Strain Behaviour

To represent the stable cyclic stress–strain behaviour, the peak tensile and compressive stress developed at half the life of the test bar (i.e., *N_f_*/2) for the two fully reversed, strain-controlled tests with total strain amplitudes of 0.3 and 0.4% at room temperature are used to develop the cyclic stress–strain curve for the A356-T7 + 0.5% Cu aluminium alloy and the fully pearlitic R260 railway steel. From Figure 12 and Figure 13, we can observe that the peak tensile and compressive stress response have stabilized at half the life of the component (*N_f_*/2) and can be used as a reasonable approximation for the development of the cyclic stress–strain behaviour of the alloy. To represent the often-smooth elastic-plastic transition of the cyclic stress–strain curves observed in most engineering metals, a Ramberg–Osgood type framework is used to model the cyclic stress–strain development of the tested alloys. Ramberg–Osgood Model for cyclic hardening:

(4) $$\varepsilon_{a}=\frac{\sigma_{a}}{E}+{\left(\;\frac{\sigma_{a}}{H^{\prime }}\;\right)}^{\frac{1}{n^{\prime }}}$$

The cyclic strain hardening exponent *n*′ and the model parameter *H*′ are used to capture the plastic hardening behaviour of the alloy. To obtain the model parameters, the test data obtained at half the life of the specimens (*N_f_*/2) for both compression and tension are calibrated against the expression shown below:

(5) $$\sigma_{a}=H^{\prime }\varepsilon_{\mathrm{pa}}^{n^{\prime }}$$

The offset yield strength σ_0_′ for the cyclic stress–strain curve is estimated at a plastic strain amplitude of 0.002 in the expression above.

**Figure 14:**
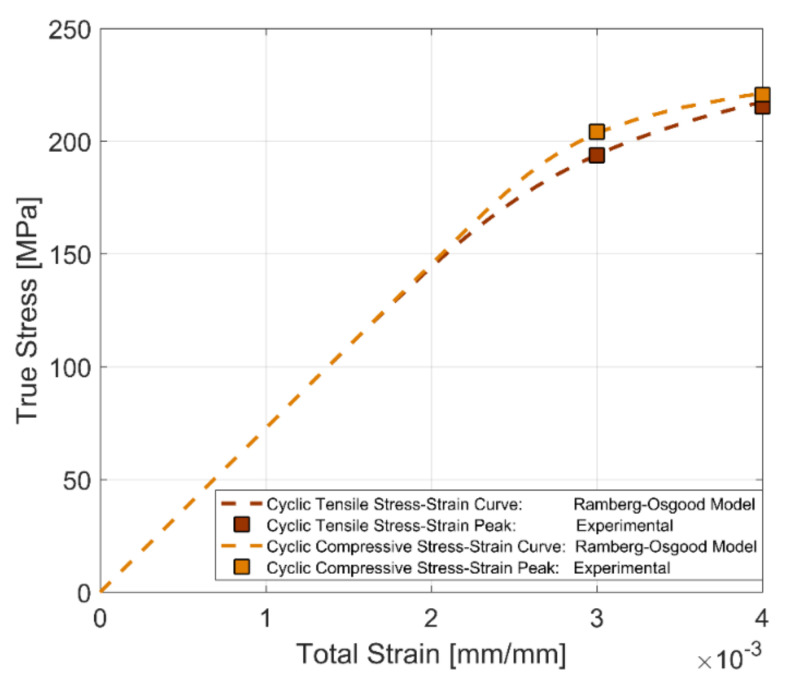
Cyclic stress–strain curve, A356-T7 + 0.5% Cu, *R_ε_* = −1.

**Figure 15:**
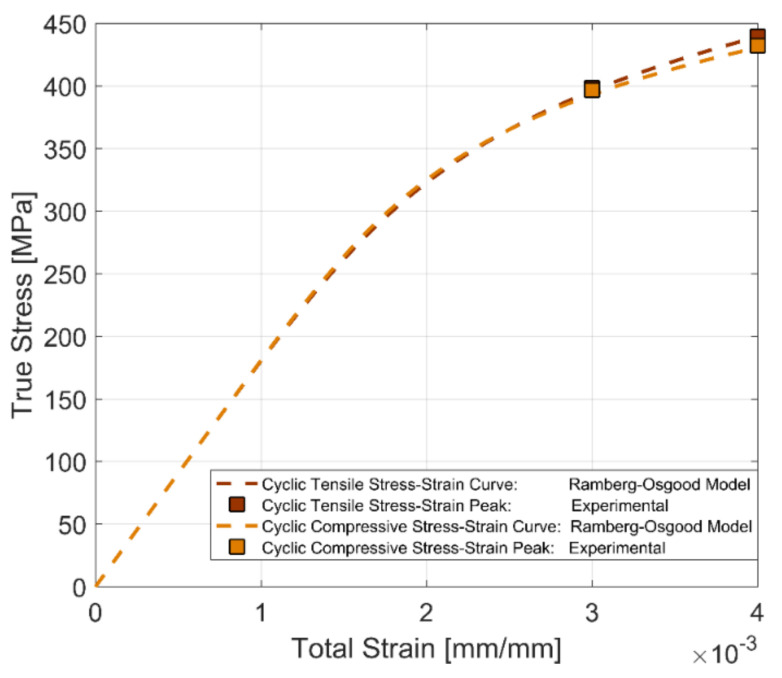
Cyclic stress–strain curve, R260 Pearlitic Steel, *R_ε_* = −1.

The cyclic stress–strain curves obtained using the stress peaks at half the life of the specimens (*N_f_*/2) for the two materials at room temperature and for completely reversed strain loads are presented in Figure 14 and Figure 15, showing the differences between the development of the tensile and compressive peak stresses in the tested A356-T7 + 0.5% Cu and R260 pearlitic steel respectively. The different asymmetry in the tension and compression peak stress development can be clearly observed for the two tested materials. The tested A356 aluminium alloy exhibits a higher stress response under compression as against the higher tensile stress response observed for the pearlitic R260 steel. The model parameters for the tensile and compressive peak stress development for completely reversed strain loading at room temperature for the two materials are summarized in Table 4.

### 3.4. Effect of Mean Strain and Temperature on Cyclic Plasticity

#### 3.4.1. Effect of Temperature and Mean Strain on Mean Stress Relaxation

**Figure 16:**
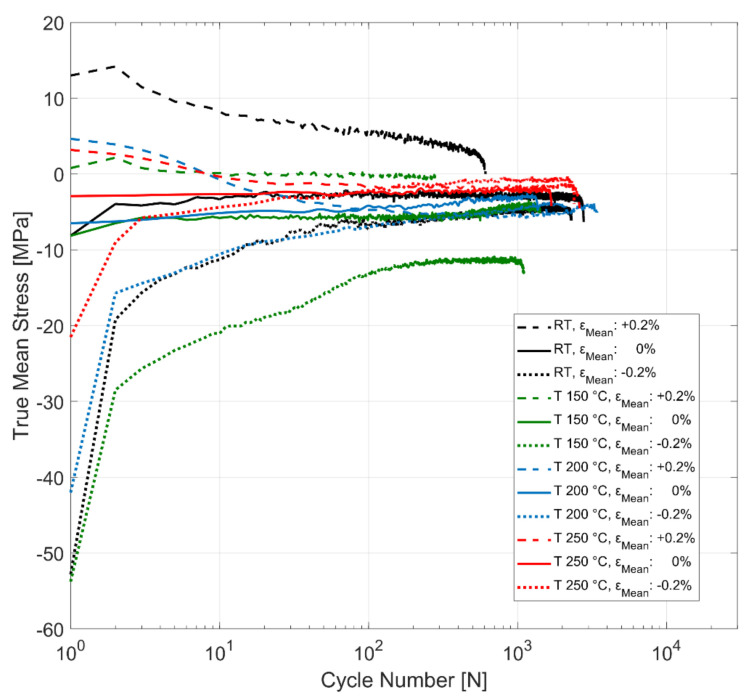
Effect of temperature and mean strain on mean stress relaxation, *ε_Amp_*: 0.4%.

**Figure 17:**
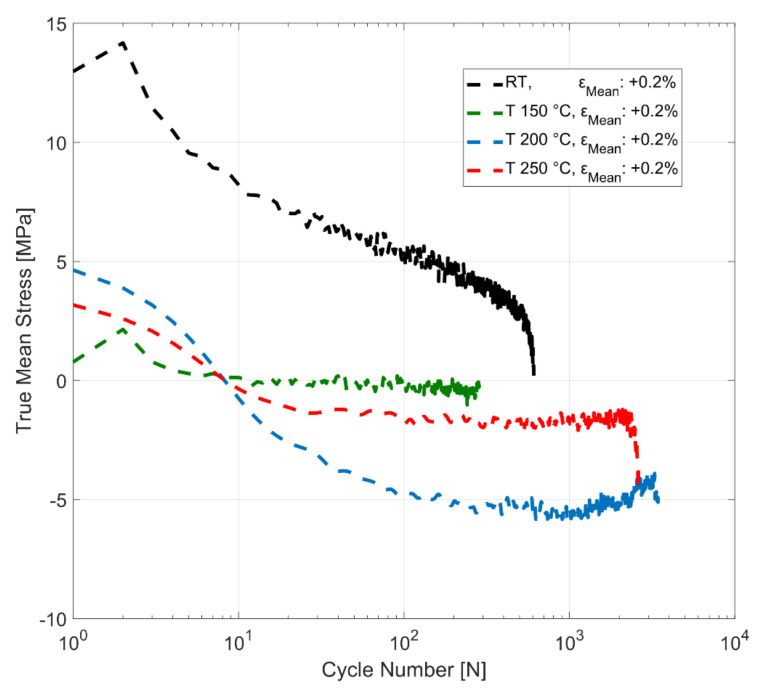
Effect of temperature and mean strain on mean stress relaxation, *ε_Amp_*: 0.4%.

The evolution of mean stress at room temperature, 150, 200 and 250 °C for strain-controlled tests with total strain amplitudes of 0.4% and mean strains of +0.2%, 0% and −0.2% is presented in Figure 16. The material develops non-zero mean stresses, indicating the peak stress asymmetry between the tension and compression for all the tested mean strain and temperature conditions. The stress asymmetry is greater at room temperature than at higher temperatures for all the load conditions. The material exhibits mean stress relaxation for the tests run with both the tensile and compressive mean strains of +0.2% and −0.2% for all the tested temperatures. The tests with 0% mean strain develop compressive mean stress for all the tested temperatures that stabilizes within the initial few cycles and remain stable with compressive mean stresses up until failure. The tests with a compressive mean strain of −0.2% exhibit greater peak stress asymmetry, with the mean stress values reaching values of around −50 MPa for room temperature and 150 °C. At higher temperatures, however, this peak stress asymmetry is less pronounced, with the test at the highest tested temperature of 250 °C exhibiting mean stress of only up to −20 MPa in the first cycle. The peak stress asymmetry between the peak tensile and compressive stresses, indicated by the mean stress development, is not as significantly affected by the tensile mean strains of +0.2% at the tested temperatures. The tensile mean stress developed for the room temperature test with a mean strain of +0.2%, for example, is about +12 MPa, compared to the −50 MPa and greater stress asymmetry observed for the room temperature tests run with a mean strain of −0.2%. Furthermore, as can be better seen in Figure 17, the tests run with the tensile mean strain of +0.2% exhibit mean stress relaxation that migrates towards compression after about 10 load cycles for the high-temperature tests at 150, 200 and 250 °C indicating the role of temperature and viscous effects in the material on mean stress development and peak stress asymmetry.

#### 3.4.2. Interplay between Cyclic Hardening and Mean Stress Relaxation

**Figure 18:**
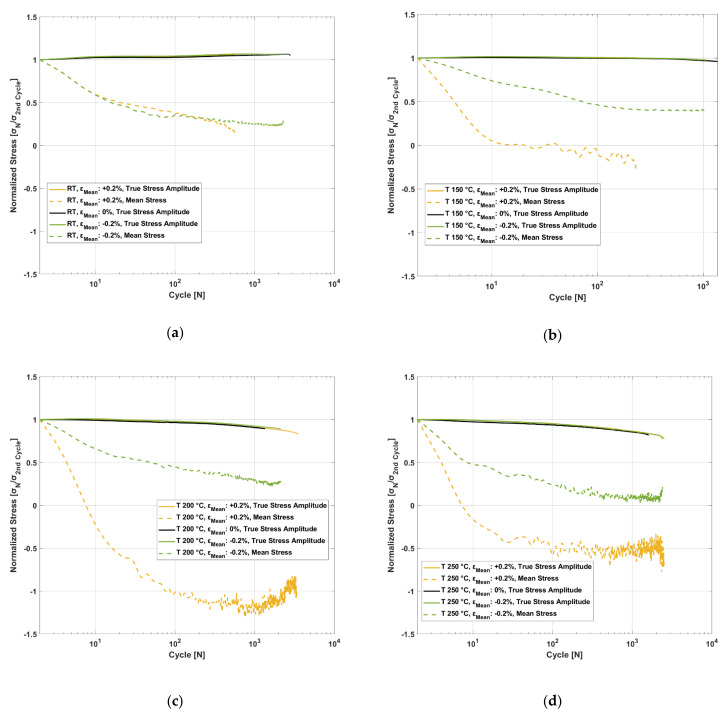
Interplay between cyclic hardening and mean stress relaxation for the cyclic strain-controlled tests with *ε_Amp_*: 0.4% and at the temperatures: (**a**) room temperature; (**b**) 150 °C; (**c**) 200 °C; (**d**) 250 °C.

The material, when subjected to cyclic strain-controlled tests with tensile and compressive mean strains, exhibit simultaneous evolution of both material hardening/softening together with mean stress relaxation. The cyclic evolution of stress amplitude and mean stress normalized against the corresponding second cycle of each test data for the various tested temperatures and mean strains is summarized in Figure 18. At room temperature, the material shows cyclic hardening for all the tested mean strains while simultaneously exhibiting mean stress relaxation for the tests with the tensile and compressive mean strains. At all the other tests at elevated temperatures, the material exhibits cyclic softening with higher temperatures resulting in higher rates of softening.

In the evolution of mean stress in strain-controlled cyclic tests with a tensile mean strain, the material exhibits a continuous mean stress relaxation throughout life. The mean stress observed at elevated temperatures changes from tensile mean stresses to zero-mean stress (symmetric stress response) and proceeds to compressive mean stresses despite the tests being run with a constant tensile mean strain throughout. At room temperature, the tensile mean stress is retained up until failure despite a continuous mean stress relaxation.

For the tests with a compressive mean strain, however, the compressive mean stress response is retained throughout the life at all temperatures despite the mean stress relaxing with progressive cyclic loads.

## 4. Discussion

Numerous research has shown the continuous variation of the elastic loading and unloading stiffness [18,38] under cyclic loading of various metallic materials. The definition of elastic modulus has a profound impact on the numerical assessment of the cyclic yield strength of a material. The stiffness of each loading segment has been evaluated for cyclic yield strength determination instead of approximating the elastic stiffness with a constant for all the loading cycles, as the stiffness has been shown to vary with plastic strain accumulation [39].

**Figure 19:**
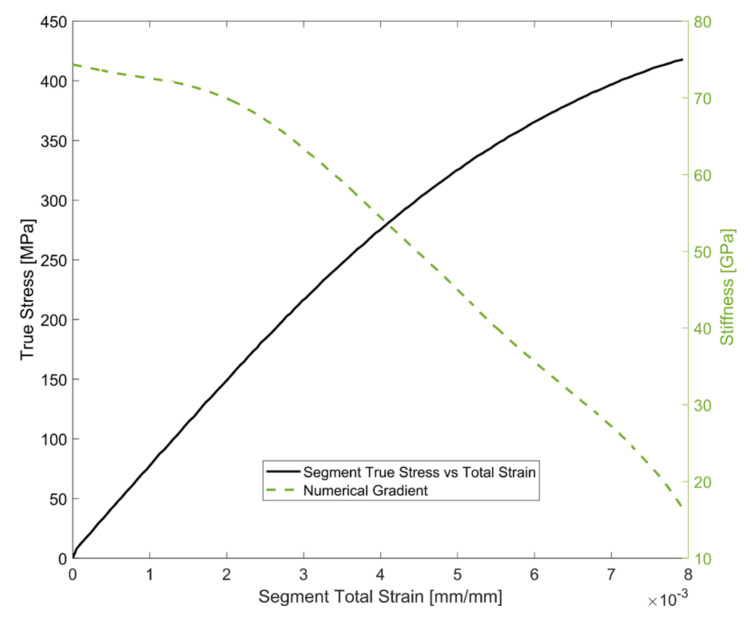
Evolution of stiffness with deformation.

The continuous numerical gradient (using unit spacing) of the true stress–strain response for a load segment of the tested A356-T7 + 0.5% Cu material at room temperature for a completely reversed strain-controlled test with the total strain amplitude of 0.4% is presented in Figure 19. The material exhibits a higher elastic stiffness than with subsequent transition of the deformation to plastic loading. The stiffness of the material can be approximated to a constant value in the elastic region for simplification despite a steady, minor reduction in stiffness with increasing strain in the ‘elastic’ part of the deformation.

**Figure 20:**
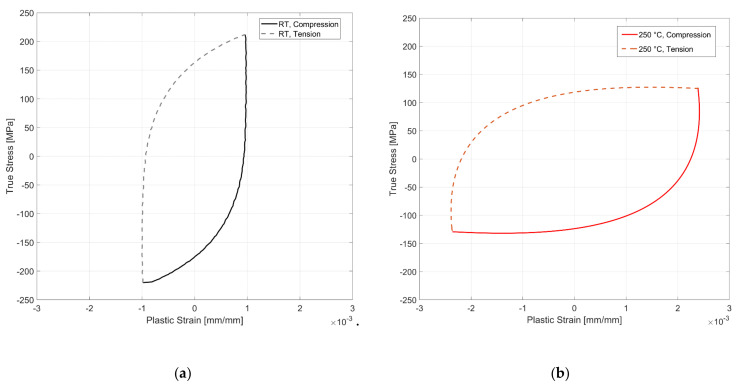
Plots showing the evolution of plastic strains determined using a linear approximation of the cyclic elastic modulus against the stress values at *N_f_*/2 for the tests at (**a**) room temperature and (**b**) 250 °C.

While a second-order elastic stiffness has been shown to work quite well for modelling the unloading stiffness in cyclically tested steel and aluminium [18,19,38,40,41], the difference observed in the yield strength determined for qualitative analysis is indiscernible for the tested A356-T7 + 0.5% Cu alloy. Figure 20 presents the evolution of plastic strains determined using a linear approximation of the elastic stress against the stress values of the half-life cycles (*N_f_*/2) at room temperature and 250 °C. The material exhibits no significant deviation of the plastic strain slope upon load reversal under ‘elastic’ unloading–loading sequence, and hence, a linear approximation of the cyclic elastic modulus is deemed an appropriate approximation for the tested material at various temperatures.

A similar yield strength asymmetry is observed with higher yield strength under compressive loading than in tensile loading even with the use of a constant elastic modulus, say from the corresponding monotonic tensile test or from the first loading segment, for the offset cyclic yield determination.

### 4.1. Yield Strength Asymmetry

Ma et al. [20] argue that constraints connected with localized strain as one of the primary factors in the tension–compression asymmetry often observed in alloys under cyclic loading in uniaxial testing. The degree of constraint experienced by the softer phase in the material is greater under compressive loading than in tension leading to the asymmetric peak stress response in the material in fully reversed cyclic tests. While this study was on single and poly crystalline copper, Mercer et al. [19] reported a similar phenomenon in dispersion and precipitation strengthened aluminium alloys. They also observed that changing the first loading from tension to compression did not affect the peak stress asymmetry, with the stress peaks developed under compression still higher than those at tension in cyclic loading. The tested A356-T7 + 0.5% Cu, being a precipitated strengthened alloy system, shows a similar peak stress asymmetry. Further, such asymmetry is observed even for the cyclic yield strength under tensile and compressive loading, as observed in Figure 9.

This stress and strength asymmetry in the tested A356-T7 + 0.5% Cu alloy, however, decreases with an increase in temperatures. Previous studies [37] have shown the tested material exhibiting softening at temperatures above 150 °C. This softening of overaged aluminium alloys strengthened by precipitation hardening is often attributed to the increase in the size of the secondary precipitates making the Orowan looping easier [42]. In the precipitation-hardened AA7075-T6 aluminium alloys investigated by Mercer et al. [19], the heat treatment process had a significant influence on the stress asymmetry observed. They argue that the asymmetry could be explained by the strain localization as a consequence of the formation of quench bands and the shearing of precipitates with regions in the material with coarser precipitates exhibiting higher strain localization.

The tested A356-T7 + 0.5% Cu shows decreasing asymmetry with an increase in the applied plastic deformation, as can be observed in Figure 14. A similar observation has been recorded for dispersion strengthened IN-9052 and IN-905XL aluminium alloys by Mercer et al. [19].

The isotropic hardening of the material often is modelled with an exponential evolution law [17,36,43] without accounting for the yield strength asymmetry [16,44,45,46] often observed in aluminium alloys [19,21]. On the basis of the evidence presented of such behaviour at all temperatures, further research into a new model that accounts for such asymmetry in aluminium alloys could potentially lead to better accuracy of the material models in numerically predicting the continuum deformation behaviour of the aluminium alloys under cyclic loading.

### 4.2. Comparison of the Number of Cycles to Failure

**Figure 21:**
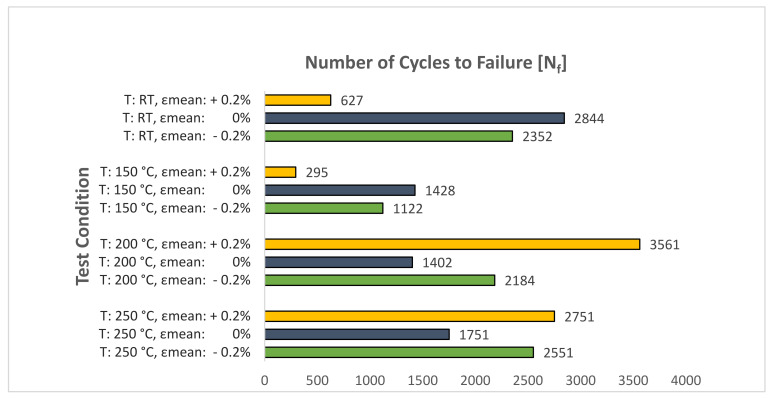
Effect of temperature and mean strain on the number of cycles to failure in A356-T7 + 0.5% Cu cast aluminium alloy.

Figure 21 presents the effect of mean strain on the number of cycles to failure at various temperatures for the tested A356-T7 + 0.5% Cu cast aluminium alloy. The material shows a dramatic reduction in the number of cycles to failure recorded in the cyclic strain-controlled tests with tensile mean strains at lower temperatures of RT and 150 °C. Similar results are reported by Houria et al. [26] in peak aged A356-T6 cast aluminium alloy, where the authors observe a reduction in the fatigue life of the material with the increase in mean stress under tensile loading at room temperature. This effect was, however, less pronounced under torsion and combined tension-torsion loadings.

For the tested A356-T7 + 0.5% Cu alloy at the elevated temperatures of 200 and 250 °C with tensile mean strains, however, such a reduction is not observed in the durability of the material, with the alloy exhibiting a similar number of cycles to failure for the tests with a zero and compressive mean strain. Similar observations are made by Azadi [25] in his studies examining the effect of mean strain on the number of cycles to failure at elevated temperatures on A356 cast aluminium alloy. The author shows the A356 material exhibiting 2316 cycles to failure on average for tests at 200 °C with a total strain amplitude of 0.4% for a completely reversed strain-controlled cyclic loading with a strain rate of 1% s^−1^. In the current study, for the identical loading scenario, the tested material failed after a similar 1402 cycles. Azadi [25], however, did not observe any effect of mean strain on the number of cycles to failure with, for example, the material exhibiting 2966 and 2991 cycles to failure for strain ratios of 0 and −12.3, respectively.

For a cast material like the tested A356-T7 + 0.5% Cu alloy that exhibits significant scatter in the number of cycles to failure [36,37], further study is required with greater sample size or with higher mean strains to draw a definitive conclusion on the effect of mean strain on the number of cycles to failure in A356-T7 + 0.5% Cu aluminium alloys.

## 5. Conclusions

To study the effect of temperature on the yield strength evolution under cyclic loading, samples of carefully extracted A356-T7 + 0.5% Cu cast aluminium alloy were tested under strain-controlled cyclic loading. The influence of tensile and compressive mean strains on the mean stress relaxation and fatigue behaviour of the material was further explored by cycling the material with a mean strain of +0.2% and−0.2%. The following conclusions could be drawn from the observed mechanical behaviour of the alloy:

- The material exhibits a non-linear cyclic hardening behaviour at room temperature. At 150 °C, the material hardens initially before quickly saturating and softening with subsequent strain load cycles. At 200 and 250 °C, the material exhibits non-linear isotropic softening.
- The material exhibits yield strength asymmetry, with higher yield strength under compressive loading than under tension. This asymmetry decreases with an increase in temperature.
- The material exhibits cyclic stress–strain asymmetry, with the peak stress response under compression higher than in tension for a fully reversed strain-controlled cyclic loading. This response is in contrast with the peak stress asymmetry observed in R260 railway steels, where the peak stress response under tension is higher than in compression for a fully reversed strain-controlled cyclic loading.
- The material exhibits mean stress relaxation for strain-controlled cyclic loading with tensile and compressive mean strains at all temperatures. The mean stress developed for tests with a compressive mean strain is higher than the corresponding tests with a tensile mean strain at all temperatures.
- The tensile mean strain has a deleterious effect on the fatigue life of the tested A356-T7 + 0.5% Cu aluminium alloy for lower temperatures up to 150 °C. At elevated temperatures of 200 and 250 °C, however, the material shows a marginal increase in the number of cycles to failure, but more tests are needed to distinguish between the scatter in the number of cycles to failure often observed in cast materials and the consequences of the tensile mean strains.

## Figures and Tables

**Table 1:** Chemical composition of the tested A356-T7 + 0.5% Cu aluminium alloy used to cast the cylinder heads in wt.%.

Si	Cu	Mg	Ti	Fe	Mn	B	Others	Al
6.8	0.53	0.35	0.12	0.10	0.07	0.0012	<0.05	Bal

**Table 2:** Chemical composition of the tested virgin R260 pearlitic steel used in rail heads in wt.%.

C	Si	Mn	P	S	Cr	N	Cu	Fe & Others
0.77	0.32	1.02	0.014	0.013	0.04	0.006	0.047	Bal

**Table 3:** Summary of extensometers used for the uniaxial tests.

Extensometer	Temperature
Instron 2620-603 axial clip-on dynamic extensometer (Instron, MA, USA)	RT and 150 °C
Epsilon 3555-010M-020 high temperature 146 axial capacitive extensometer (Epsilon Technology Corporation, Jackson, WY, USA).	200 and 250 °C

**Table 4:** Ramberg–Osgood model parameters for tensile and compressive stress response under cyclic loading.

Material	Loading	$\mathrm{Offset}\;\mathrm{Yield}\;\mathrm{Strength}\;\sigma_{0}^{\prime }\;[\mathrm{MPa}]$	H′ [MPa]	Cyclic Strain Hardening Coefficient n′
A356-T7 + 0.5% Cu	Tension	233	443	0.1033
	Compression	230	323	0.0542
R260	Tension	456	1205	0.1565
	Compression	443	1035	0.1365

## Data Availability

The raw/processed data required to reproduce these findings cannot be shared at this time as the data also form part of an ongoing study.
